# Teaching AI, teaching bias: addressing gender inequality in public health education

**DOI:** 10.3389/fpubh.2026.1952627

**Published:** 2026-10-14

**Authors:** Andrea Dietrich, Karolina Czok, Karina Ilskens

**Affiliations:** 1 Center for Cognitive Interaction Technology (CITEC), Bielefeld University, Bielefeld, Germany; 2 Chair of Digital Public Health, Department of Social Sciences, Faculty of Arts and Humanities, University of Siegen, Siegen, Germany; 3 Faculty of Health, Institute for Educational and Health-Care Research in the Health Sector, Hochschule Bielefeld - University of Applied Sciences and Arts Bielefeld, Bielefeld, Germany

**Keywords:** algorithmic bias, artificial intelligence, bias, education, gender, gender bias, health equity, public health

## Abstract

Artificial intelligence increasingly shapes healthcare, public health and health professions education. However, predictive and generative systems can reproduce or amplify longstanding inequalities embedded in data, institutions and established knowledge structures. Public health education therefore needs to connect technical approaches to identifying and mitigating algorithmic bias with their broader social, population health and institutional contexts. This conceptual article presents the sociotechnical Bias literacy for public health education framework (SBL-PHE), developed through a theory-informed conceptual synthesis drawing on conceptual framework analysis. A focused supplementary literature search in PubMed/MEDLINE, Scopus, ERIC and the ACM digital library was conducted to examine and refine its theoretical foundations. An evidence-informed composite case of AI-supported cardiovascular risk assessment traces algorithmic gender bias from knowledge production and data representation through modeling and implementation to monitoring. The framework comprises five interconnected competency dimensions concerned with recognizing, explaining, evaluating, addressing and reflecting on algorithmic bias. For master’s-level public health education, these dimensions are linked to learning objectives, learning activities, assessment methods and a prospective evaluation strategy. Sociotechnical Bias literacy is proposed as a foundational competency for public health professionals who use or interpret AI. Sociotechnical fluency describes an advanced, role-dependent capability, particularly for responsibilities in implementation, evaluation, translation and governance. SBL-PHE provides a theoretically grounded framework for integrating technical understanding, health equity, professional judgment and institutional responsibility into public health education. The framework and its proposed educational application have not yet been empirically validated.

## 1 Introduction

Artificial intelligence is increasingly integrated into healthcare, public health and health professions education. Predictive systems support risk stratification, screening and resource allocation, while generative systems can produce educational materials and organize health-related information. Public health education must prepare professionals to assess both their potential uses and their limitations (1, 2). This includes examining how such systems represent sex and gender, which populations’ experiences inform the data used to develop them, and how their use may affect health equity (3).

AI systems learn from data shaped by research priorities, documentation practices and unequal access to healthcare. These conditions can introduce distortions into models and their outputs (4, 5). Gender bias provides a particularly relevant perspective on this process. Women have historically been underrepresented in cardiovascular trials, while educational materials can reproduce stereotypical expectations about patients and health professionals (6, 7). Numerical representation alone does not resolve the problem. In the cardiovascular prediction study by Li et al. (8), women constituted 64.5% of the 109,490-person development cohort, yet the evaluated models showed disparities disadvantaging women. Assessment must therefore also consider outcome definitions, feature distributions and model decisions, without assuming that any single mechanism explains every disparity.

The consequences extend beyond individual encounters. A system deployed across a health service can repeatedly apply the same decision rule, affecting access to prevention, assessment or treatment across its population. Such repeated, scaled effects make algorithmic bias a public health concern in its own right (5). It also directs attention to institutional responsibilities for procurement, local validation, implementation and monitoring, alongside the responsibilities of individual professionals (9).

Technical approaches to identifying and mitigating bias remain essential. They cannot, on their own, determine which differences are clinically justified or which distribution of benefits and burdens is acceptable (10, 11). Nor does the formal presence of a human in the decision loop ensure effective oversight. Professionals require relevant information, contextual understanding and the authority to question or interrupt recommendations (12, 13).

AI literacy already encompasses critical evaluation, communication and understanding of AI, as well as its practical use (14). Building on this foundation, public health education needs to connect the analysis of bias with population health, institutional responsibility and health equity. Such an orientation aligns with the emphasis on evidence-informed practice, communication and collaboration in the WHO public health competency framework (15).

This article introduces the Sociotechnical Bias Literacy for Public Health Education Framework (SBL-PHE). Within this framework, Sociotechnical Bias Literacy denotes the ability to recognize, explain, evaluate, address and reflect on algorithmic bias in its technical and social context. Sociotechnical Fluency describes a more advanced capacity to translate between technical evidence, professional knowledge and institutional decisions. It is a role-dependent educational horizon, particularly relevant to implementation, evaluation and governance, rather than a uniform requirement for all graduates. These proposed concepts are developed in section 2.4. An evidence-informed composite case of cardiovascular risk assessment connects the framework to master’s-level public health education. The case focuses on sex- and gender-related disparities while also considering how these interact with age, socioeconomic circumstances and access to care.

## 2 Theoretical foundations

The SBL-PHE Framework rests on the premise that algorithmic bias cannot be understood solely as a technical property of a model or solely as individual prejudice. It arises through relationships between knowledge production, data collection, modeling, institutional decision-making and societal power. Public health education must therefore consider technical mechanisms, the distribution of health effects and professional responsibility together.

### 2.1 AI systems and concepts of bias

For the purposes of this article, AI refers to computational systems that produce classifications, predictions, recommendations or generated content. The focus is on systems that learn from data. Predictive or discriminative models estimate outcomes or distinguish categories, whereas generative models produce content such as text or images. These functions can coexist within a system, so the distinction does not define mutually exclusive technologies.

Predictive and discriminative models estimate labels, probabilities or risks from input data. In healthcare, they can support cardiovascular risk prediction, screening, triage or resource allocation. Their performance depends first on how populations, outcomes and predictors are represented. Other determinants include the construction of labels and proxies, the choice of objectives and thresholds, and validation for the intended population and application context. Group differences may remain concealed when only average performance is reported. Assessment must therefore address both average performance and the populations, labels and decision rules on which it depends (4, 8, 16).

Large language models are typically pretrained through self-supervised learning and may subsequently undergo supervised fine-tuning or preference-based alignment. Self-supervision does not eliminate human choices about the collection, filtering and composition of training corpora. Human annotation can also contribute during later training or evaluation. Bias assessment must therefore identify the particular development stage and application being examined. Nemani et al. (17) review approaches to detecting and mitigating gender bias in transformer models and highlight limitations in metrics, datasets and evaluation standardization. Their review provides a methodological overview rather than evidence that one assessment method is sufficient for all systems.

Generative systems also raise questions about how people interpret outputs. Carnat (18) examines automation bias and anthropomorphism in relation to generative language models. This conceptual and legal analysis draws attention to conditions under which fluent outputs may receive undue trust; it is not an empirical demonstration that all users or clinical applications exhibit the same response.

Bias has distinct meanings across these discussions. Statistical bias concerns systematic estimation error, while inductive bias refers to assumptions that support generalization beyond observed examples (19). Neither is synonymous with discrimination. In sociotechnical analysis, attention instead centers on unjustified disadvantage, distortion and stereotypical representation arising through data, models and their contexts of use (20). Sex-related biological characteristics and gender-related social roles and relations can interact, but should not be treated as interchangeable variables (3).

Selection, generalization and differentiation between groups are not inherently unfair. Accounting for sex-related differences can be clinically appropriate when supported by relevant evidence and used to improve diagnosis or treatment (3). Such differentiation becomes problematic when sex or gender serves as an unjustified proxy, relevant heterogeneity is overlooked, or recommendations reproduce inequities without adequate clinical justification (10). The question is therefore not simply whether a model differentiates, but whether the distinction is justified for its intended purpose and consequences.

The distinction can be illustrated by the gender-neutral Turkish sentences “O bir doktor” and “O bir hemşire.” Rendering them as “He is a doctor” and “She is a nurse” introduces gender information absent from the source. Although women may constitute a numerical majority in nursing, this does not justify assigning a gender to an unspecified individual. Gender-neutral translations are possible. Repeated stereotypical assignments can reinforce expectations about who belongs in a profession. This example illustrates the occupational stereotyping examined in machine translation research (21). Related research identifies gendered occupational associations in word embeddings (22), although these representations and translation systems involve different technical mechanisms.

### 2.2 Gender bias in sociotechnical knowledge production

Training data are produced, selected, documented and annotated within institutions. Research priorities determine which questions receive funding, clinical routines influence which symptoms and diagnoses are recorded, and classification systems determine which experiences become measurable. Datasets therefore do not represent social reality neutrally. They reflect decisions about relevance, categories, evidence and representation (23–26).

Formalizing health experiences as standardized variables can leave social roles, biography and experiential knowledge incompletely represented. Technical optimization does not remove these limitations. A model may minimize average error while performing less well for particular groups, or rely on shortcuts that work in the development data but fail under different conditions. Shortcut testing offers one way to investigate such behavior in medical AI (27). Determining why an association arose and whether it is appropriate also requires knowledge of the setting in which data were produced.

Gender-related gaps are evident in educational materials. Dijkstra et al. (28) examined medical textbooks and identified shortcomings in sex- and gender-related content. In undergraduate medical teaching and assessment materials in Geneva, Arsever et al. (7) analyzed 955 eligible clinical vignettes and found stereotyped patterns in occupations, caregiving roles and the portrayal of health professionals. These findings concern particular educational materials, but illustrate how professional expectations can be reproduced through teaching. Their reuse as training material or generated teaching content is a plausible pathway requiring scrutiny, rather than an outcome established by these studies.

Clinical evidence also requires careful differentiation. Wilford et al. (29) reviewed gender bias in musculoskeletal pain management and reported patterns in which men more often received exercise recommendations for back pain and laboratory testing, while women more often received recommendations for psychological treatment and counseling. These heterogeneous findings do not establish uniform disadvantage in every encounter. Le Boudec et al. (30) studied medical students in an examination with standardized patients and identified sex-related differences in aspects of their clinical approach. This evidence concerns a simulated assessment setting, rather than observed inequalities in routine patient outcomes.

Research priorities may shape the evidence available for teaching and modeling. Cook et al. (31) reported that 64 of 82 single-gender alcohol research articles from Australia and Aotearoa New Zealand focused on women, corresponding to 78%. The distribution alone does not demonstrate bias; its interpretation depends on the research questions and disease burden. Van Hagen et al. (32) examined 38 public health research proposals and identified limitations in how sex and gender were considered, showing why scrutiny should begin during research planning.

Amaya-Santos et al. (33) found that 22 of 43 included studies on healthcare AI frameworks explicitly considered sex or gender, corresponding to 51.2%. This identifies a limitation in the reviewed literature rather than establishing the completeness of any alternative framework. SBL-PHE therefore proposes examining gender alongside other social positions and institutional conditions. Intersectionality concerns how these relationships combine to shape disadvantage; it is not achieved simply by adding demographic variables or comparing women with men (34, 35).

Together, these findings support a sociotechnical understanding of algorithmic fairness in which data, models, organizations and professional practices must be examined in relation to one another (11, 36). We use bias laundering as a descriptive term for the obscuring of historical and social origins when patterns are encoded and returned as apparently neutral algorithmic outputs. The term describes the proposed interpretation of this process, rather than a distinct experimentally established mechanism.

Responsibility can also become obscured when decisions are presented as technologically predetermined. Rubel et al. (37) analyze this problem through the concept of agency laundering. For the present framework, this reinforces the need for traceable data practices and identifiable decision-makers (38).

Figure 1 brings together the preceding discussion of how historically shaped knowledge enters data and models. It illustrates bias laundering, whereby algorithmic processing can lend an appearance of objectivity to existing biases. When outputs are accepted as neutral evidence, these biases can feed back into professional judgment, educational content, institutional practices and future data generation.

**Figure 1:**
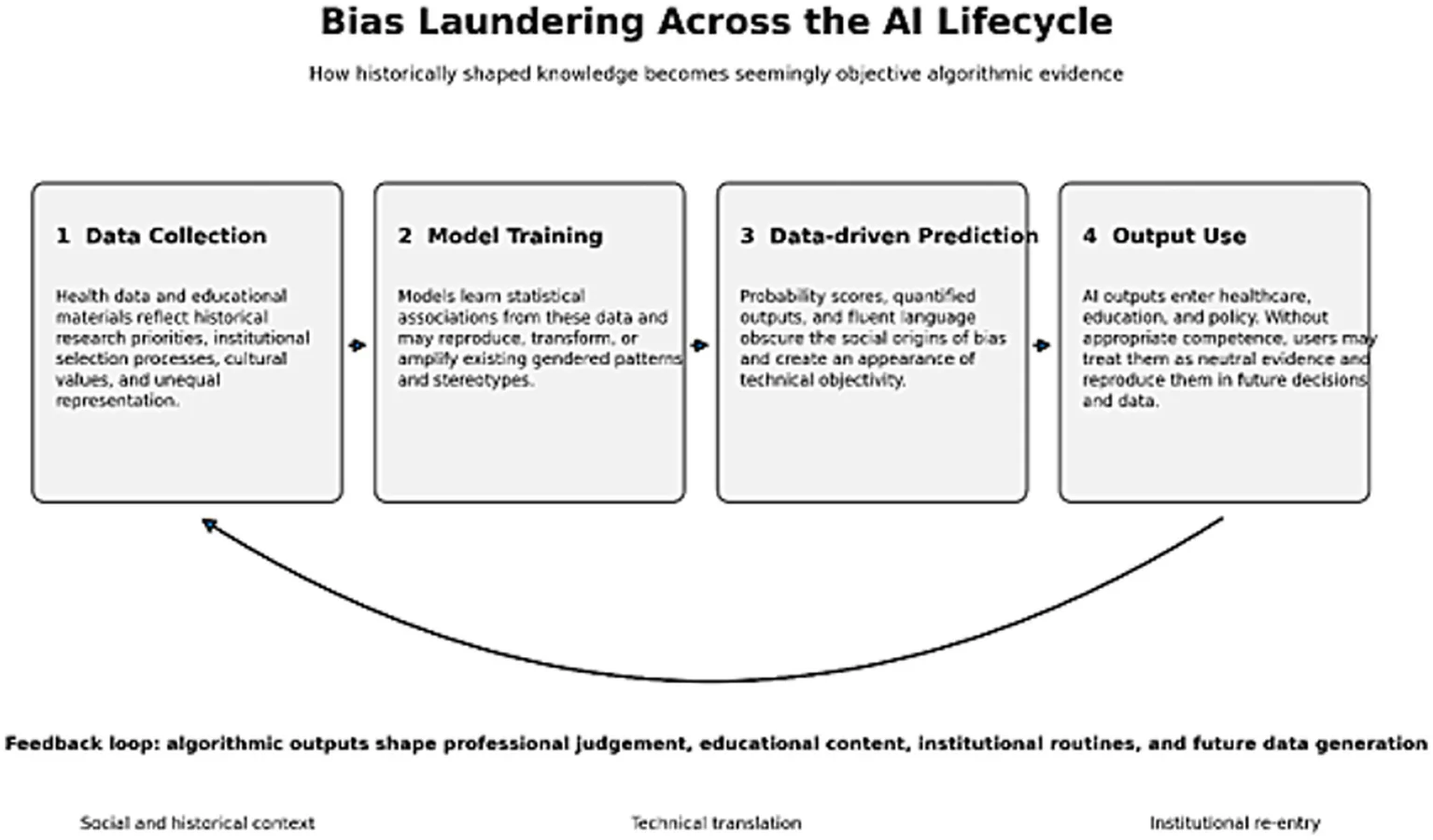
Bias laundering across the AI lifecycle. Historically shaped data and knowledge structures enter model training and are transformed into seemingly objective algorithmic outputs. When treated as neutral evidence, these outputs can reinforce existing biases in professional judgment, educational content and institutional practices, while shaping future data generation.

### 2.3 Technical assessment and human oversight across the AI lifecycle

Technical assessment begins with the data foundation. Data profiling can identify differences in selection, missingness, label distributions and the availability of relevant variables. Documentation should record provenance, collection conditions and limitations. Data Statements were developed specifically for language datasets; their purpose should be distinguished from the wider documentation needed for clinical datasets (39). Dataset accountability also requires attention to decisions across the development lifecycle (38). Neither dataset size nor demographic balance alone demonstrates fitness for an intended clinical use.

Methods at the model level must match the architecture and the question being asked. The Word-Embedding Association Test assesses associations between sets of words and attributes in embedding spaces (40). It does not directly measure clinical discrimination or the behavior of every generative model. BertViz visualizes attention in transformer models (41), while research on implicit knowledge extraction considers how information encoded in neural networks can be examined (42). These approaches can inform scrutiny of representations, but attention patterns or encoded associations do not, on their own, establish why a particular decision occurred.

Output assessment examines sensitivity, specificity, predictive values, calibration and error patterns in relevant subgroups. Different fairness criteria address different questions. Equal opportunity concerns true-positive rates, whereas demographic parity concerns rates of positive decisions. Under differing outcome prevalence, certain fairness requirements cannot generally be satisfied together; the assumptions and exceptions must be specified (43, 44). The implications are developed in the cardiovascular case. Selecting a criterion requires both statistical analysis and a justification of which consequences matter in the intended setting.

LIME and SHAP offer feature-attribution approaches that can help interpret model predictions, but their explanations do not establish causality or certify fairness (45, 46). Model Cards document intended uses, evaluation and limitations, including subgroup performance where appropriate (47). Such documentation supports informed assessment; it does not replace validation or resolve normative disagreements.

After deployment, changes in populations, documentation and care practices can alter performance. Continuous auditing connects repeated assessment with responsibilities for responding to findings (48). In healthcare, an audit must also examine the system’s use and effects within its institutional setting (9).

Table 1 summarizes these analytical levels, complementary methods and their proposed educational relevance.

**Table 1:** Analytical levels of the AI lifecycle, assessment methods and their relevance to SBL-PHE.

Lifecycle phase	Analytical focus	Assessment and documentation methods	Relevance to SBL-PHE
Data and evidence	Representation, missing observations, outcome definitions and label construction	Data profiling; missingness and label analysis; provenance documentation; Data Statements for language datasets (38, 39)	Recognize representation gaps and explain how data practices shape the evidence available to a model.
Model representations	Learned associations and encoded information, assessed with methods appropriate to the architecture	WEAT for word embeddings; representation analysis and probing; BertViz for transformer attention (27, 40–42)	Explain associations and distinguish evidence about representations from evidence about the causes of a decision.
Outputs and application	Subgroup performance, fairness priorities and the interpretation of predictions	Subgroup sensitivity, specificity, predictive values and calibration; fairness metrics; LIME and SHAP; Model Cards (43–47)	Evaluate disparities and trade-offs and justify the interpretation of outputs in their intended context.
Implementation and monitoring	Performance in the local setting, changes over time and capacity to respond	Local validation; repeated subgroup and error analysis; drift and incident monitoring; continuous auditing and documented escalation (9, 13, 48)	Address identified risks, assign responsibilities and reflect on uncertainty and the limits of human oversight.

Authors’ conceptual synthesis based on the sources cited in the respective table rows. Educational mappings are proposed applications, not measured learning outcomes. WEAT assesses associations in word embeddings; attention visualization and feature attribution do not establish causal explanations or demonstrate fairness. Model Cards and Data Statements document systems and datasets but do not themselves validate them. Methods should be selected according to the system type and intended use. WEAT, Word-Embedding Association Test; LIME, Local Interpretable Model-Agnostic Explanations; SHAP, SHapley Additive exPlanations.

Human-in-the-loop involves more than placing a person at the end of an automated decision. Oversight can operate during development, validation, application and monitoring. Its effectiveness depends on when a person can intervene, what information is available, and whether sufficient time, expertise and decision-making authority are provided. Interface design, workload and automation bias also influence whether a recommendation is scrutinized or merely confirmed (13, 49).

Carnat (18) analysis further indicates why oversight arrangements for generative systems should consider automation bias and anthropomorphism. In SBL-PHE, the educational implication is that professionals must be able to question plausible outputs and understand when human intervention is warranted. This is a proposed application of the analysis, rather than an evaluated learning effect.

Without adequate model understanding and contextual knowledge, the human in the loop can become the final layer of confirmation for a flawed system rather than an effective safeguard. An individual cannot compensate for technical and institutional deficiencies alone. Organizations must assign responsibilities, document deviations and provide a safe fallback pathway. Audit findings must reach a body with the authority to initiate changes or restrict use. Human oversight, continuous auditing and governance therefore work together to distribute responsibility across the system lifecycle (9, 12, 48).

### 2.4 Sociotechnical bias literacy and sociotechnical fluency

Long and Magerko (14) conceptualize AI literacy in terms that include critical evaluation and effective communication and collaboration with AI. SBL-PHE builds on this broad foundation by specifying competencies for examining algorithmic bias in public health. Its emphasis on contextual judgment is informed by pedagogical work on learning with AI and on gender equity in health professions education (15, 50, 51). These sources inform the approach; they do not establish or validate the five SBL-PHE dimensions.

Within the proposed framework, Sociotechnical Bias Literacy denotes the ability to recognize algorithmic bias, explain its origins, evaluate its consequences, develop appropriate responses and reflect on professional responsibility. The five dimensions are the authors’ conceptual synthesis. They apply to professionals who use, communicate or interpret AI-supported information, with the depth of competence determined by professional role, decision-making authority and potential harm.

Sociotechnical Fluency extends this foundation through the ability to translate between model logic, professional knowledge, organizational conditions and population-level consequences. It is particularly relevant to people responsible for procurement, implementation, evaluation and governance. Literacy enables a professional to identify and question an inadequately justified recommendation; fluency additionally enables that person to connect the technical evidence with operational decisions and coordinate an appropriate response across professional groups.

Public health professionals do not all need to conduct technical audits themselves. They do need sufficient understanding to formulate relevant questions, interpret audit findings and recognize when specialist expertise is required. This educational orientation connects technical scrutiny with the communication, collaboration and evidence-informed practice emphasized in the WHO public health competency framework (15). Figure 2 illustrates the proposed translational function of Sociotechnical Fluency between algorithmic representations and the public health context. Empirical evaluation is required to establish how reliably these proposed competencies can be taught and assessed.

**Figure 2:**
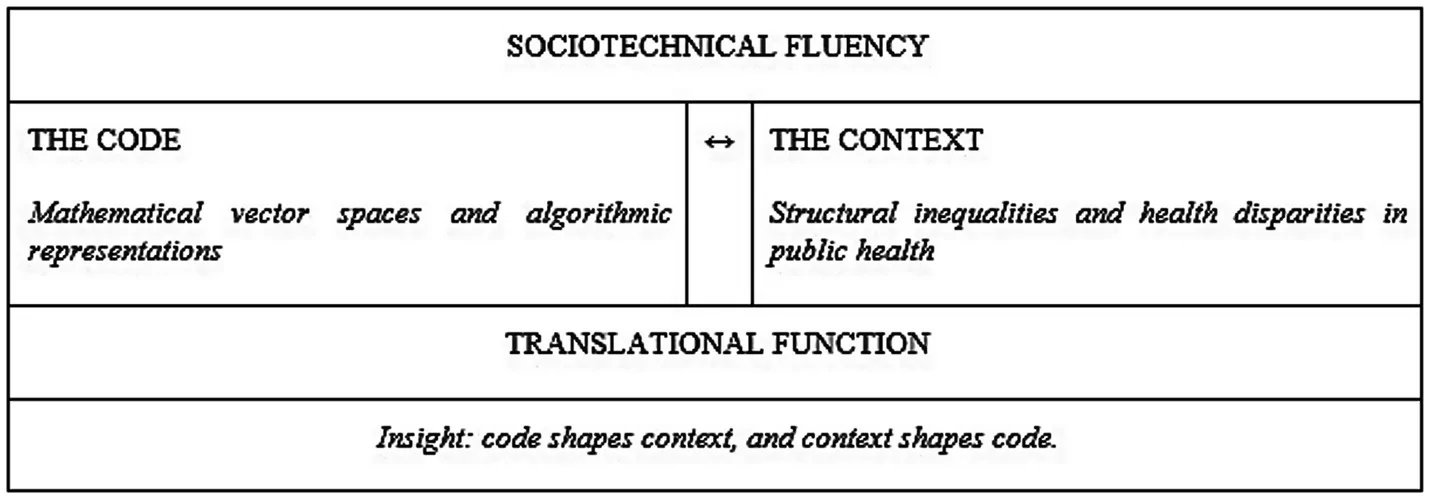
Sociotechnical fluency. Translating between mathematical and algorithmic representations (“The Code”) and structural inequalities and health disparities (“The Context”) connects technical understanding with public health judgment. The bidirectional relationship emphasizes that code shapes context and context shapes code.

Figure 2 illustrates this translational function between algorithmic representations and the public health context.

## 3 Methods and approach

### 3.1 Conceptual research design and epistemic scope

The SBL-PHE Framework was developed through a theory-informed conceptual synthesis connecting research on algorithmic bias, sociotechnical systems, public health competencies and higher education pedagogy. The approach draws on the identification, comparison and integration of concepts described in Jabareen (52) Conceptual Framework Analysis. Jaakkola (53) account of theory synthesis informs the explicit selection of perspectives and the explanation of their respective roles. These approaches provide methodological guidance for the present synthesis; the study does not report a complete implementation of Jabareen’s eight-phase procedure.

SBL-PHE organizes relationships between bias mechanisms, professional competencies and educational applications. It is intended to support understanding and curriculum design rather than predict learning outcomes. Its coverage, educational effectiveness and transfer to professional practice require empirical evaluation.

The initial framework brought together four conceptual domains comprising bias across the AI lifecycle, sociotechnical and feminist perspectives on data and fairness, public health competencies, and AI literacy and higher education pedagogy. A focused supplementary literature search was subsequently conducted to examine and refine these foundations. The cardiovascular literature provided the empirical context for a composite teaching case. This supplementary work strengthened the original framework without retrospectively constituting its sole developmental basis.

### 3.2 Focused supplementary literature search and selection

The literature informing the initial framework was assembled iteratively during manuscript development. During revision, this existing literature base was supplemented through focused database searches and the examination of additional relevant publications. The documented database searches were conducted on 30 August 2026 in PubMed/MEDLINE, Scopus, ERIC and the ACM Digital Library. Subsequent source verification and the consideration of additional publications continued until 8 September 2026. The database search date therefore identifies the retrieval stage rather than the duration of literature assessment and conceptual synthesis. Database-specific queries used text terms and, where included in the recorded strategy, controlled vocabulary. Three search clusters addressed AI literacy and education in public health and the health professions; algorithmic sex and gender bias in cardiovascular prediction and the clinical evidence informing the case; and sociotechnical fairness, governance, human oversight and auditing. These retrieval clusters served a different purpose from the conceptual domains used to organize the synthesis.

Publication windows differed between search strands. Education searches generally began in 2019, with ERIC using its since-2017 filter. Governance and auditing searches generally began in 2015, while searches on cardiovascular trial representation and symptom presentation extended back to 2000. PubMed searches used an end date of 30 August 2026; interfaces such as Scopus used publication-year restrictions including 2026. Foundational works and official frameworks were identified separately. The combination of database and supplementary retrieval is informed by evidence on searching complex literatures (54). Iterative attention to conceptual relevance also draws on Dixon-Woods et al. (55), without claiming a full Critical Interpretive Synthesis. Supplementary source verification continued through 7 September 2026.

The retained archive contains 2,453 database records, comprising 1,087 from PubMed/MEDLINE, 1,085 from Scopus, 133 from ERIC and 148 from the ACM Digital Library. Together with 38 supplementary records, the archive contains 2,491 records. Matching bibliographic identifiers and normalized titles consolidated 404 duplicate records into 2,087 record groups. These are retrieval and record-linkage counts. They do not represent the number of publications included in the conceptual synthesis. The search log, retained records and linkage information were documented as part of the conceptual synthesis process.

Source selection for conceptual synthesis was purposive. Priority was given to publications contributing a relevant bias mechanism, empirical finding, competency concept, educational approach or governance implication. Technical performance optimization alone, or the use of AI solely as a generic teaching tool, was insufficient for targeted selection without a substantive connection to the framework. A preliminary shortlist of 80 candidates supported orientation. It was not a completed full-text selection, and records outside the targeted selection remain unassessed rather than counted as relevance exclusions. Exhaustive screening and conceptual saturation are not claimed.

Targeted extraction was documented for 26 core sources supporting methodological design, educational foundations, the cardiovascular case, fairness concepts and institutional responsibility. Accessible full texts or relevant book sections were examined for their contribution to the synthesis. The evidence matrix records source identity and type, the consulted passage or section, context, AI type and lifecycle relevance where applicable, the principal finding or concept, its proposed use in SBL-PHE and limits on interpretation. Extraction was AI-assisted and was not independently duplicated. This documented core is distinct from the full manuscript bibliography; additional cited sources were used to substantiate specific statements. The matrix separates findings reported by a source from educational applications proposed for SBL-PHE.

### 3.3 Iterative derivation and evidence mapping

The conceptual mapping was organized around four analytical questions. These concerned where bias can arise or become visible, which technical and institutional mechanisms are involved, what consequences may follow for populations and health equity, and what professionals need to understand and do in response. Relevant concepts were compared across the four conceptual domains and related to the cardiovascular case.

Lifecycle analyses supplied the technical and temporal structure. Sociotechnical and feminist perspectives drew attention to the categories, values and institutional arrangements through which data become actionable knowledge. Public health competencies supplied the population and professional context, while educational theory informed the translation into learning objectives and assessment. The cardiovascular literature was used to construct a continuous illustrative case. Findings from different studies were retained with their original contexts and were not treated as observations from one shared system or population.

This mapping organized professional responses into five interconnected SBL-PHE competency dimensions concerned with recognizing, explaining, evaluating, addressing and reflecting on algorithmic bias. Recognition and explanation connect potentially consequential patterns with their mechanisms. Evaluation considers the evidence and its implications, while addressing concerns proportionate action. Reflection examines uncertainty, assumptions and responsibility. The dimensions are the authors’ conceptual synthesis. They are not categories statistically derived from the retrieved records or a validated sequence of learning stages.

Learning objectives, activities and assessment tasks were linked through constructive alignment (56). The distinction between factual, conceptual, procedural and metacognitive knowledge was informed by Krathwohl (57) account of the revised taxonomy. Miller’s (58) distinction between knowing, knowing how, showing how and doing informed the separation of knowledge assessment, demonstrated performance and professional practice. These educational perspectives guide the proposed learning design without establishing a one-to-one correspondence with the five SBL-PHE dimensions. Learners may revisit any dimension as new evidence or contextual information becomes available.

Table 2 summarizes the conceptual domains and the cardiovascular evidence, provides representative sources and identifies their proposed contributions. A source-level evidence matrix was used to document the 26 sources selected for targeted extraction. Together, these mappings make the conceptual reasoning visible while preserving the distinction between literature-supported findings and proposed educational design.

**Table 2:** Conceptual foundations and their proposed contributions to development of the SBL-PHE framework.

Conceptual domain and representative sources	Guiding question	Role in framework development	Proposed contribution to SBL-PHE competencies
AI lifecycle and technical bias (4, 9)	Where can bias arise, change or become visible from data production to monitoring?	Defines lifecycle phases and technical questions for analysis.	Supports recognizing bias and explaining its mechanisms.
Sociotechnical fairness and data feminism (11, 25, 26)	How do data categories, such as recorded sex or gender, diagnoses and outcome labels, interact with institutions, values and power?	Examines how categorization and institutional practices shape evidence, responsibility and governance.	Supports evaluating consequences, addressing risks and reflecting on assumptions and responsibility.
Public health competencies and health equity (35, 68)	How do systems deployed at scale affect populations, access to care and the distribution of resources?	Connects technical findings with population health and professional responsibilities.	Positions health equity and interacting social circumstances as considerations across the five competency dimensions.
AI literacy and higher education pedagogy (14, 57, 58)	How can conceptual understanding be translated into learning activities and assessable performance?	Links learning objectives, activities and assessment with professional roles.	Supports the educational translation and assessment of the five competency dimensions.
Cardiovascular evidence on sex and gender (3, 8, 16)	How can representation, outcome definitions and model decisions contribute to sex- or gender-related disparities?	Provides the evidence-informed composite case used throughout the lifecycle analysis.	Integrates recognizing, explaining, evaluating, addressing and reflecting on algorithmic bias in the case.

Authors’ theory-informed synthesis. Data categories refer to the definitions and coding practices used to represent people and outcomes, rather than classification algorithms. The five SBL-PHE competency dimensions are recognizing, explaining, evaluating, addressing and reflecting on algorithmic bias. Their mapping to the literature is a proposed educational interpretation, not evidence of measured learning outcomes. The cardiovascular evidence is an application domain alongside the four conceptual domains.

## 4 Results

The conceptual synthesis yielded the SBL-PHE competency framework and its proposed application through a continuous cardiovascular teaching case. The results presented here comprise the conceptual framework, educational design and prospective evaluation approach. The framework is intended for educators, program directors and institutions developing or revising AI-related teaching. Its illustrative learner group comprises master’s students in Public Health, Population Health or Health Sciences, with introductory knowledge of epidemiology, research methods and statistics. Programming experience is not required. Interprofessional participation can bring medical, nursing, data science and organizational perspectives into the proposed activities.

### 4.1 The SBL-PHE competency framework

Building on the mapping described in Chapter 3, SBL-PHE connects technical scrutiny with professional judgment about how AI affects populations. Analysis across the AI lifecycle means following the development and use of a system from knowledge and data production through modeling and decision-making to implementation and monitoring. The framework translates the questions arising at these stages into five interconnected competency dimensions concerned with recognizing, explaining, evaluating, addressing and reflecting on algorithmic bias.

Recognizing bias involves identifying potentially consequential patterns in evidence, data, model documentation or outputs. Explaining bias requires tracing how such patterns may arise through measurement, labels, model objectives or institutional practices. Evaluation then considers the strength of the evidence, uncertainty and the distribution of benefits and harms. Addressing bias involves proposing proportionate technical or organizational responses, including escalation or a decision against deployment. Reflection concerns the assumptions, professional roles and limits of knowledge that shape each judgment. These dimensions overlap. For example, evaluating a subgroup disparity may reveal an outcome definition that requires renewed examination of the data.

Table 3 connects each dimension with knowledge, skills and professional dispositions. The framework brings together AI literacy, sociotechnical analysis and public health competencies rather than treating bias as a technical defect that can be understood independently of context (14, 15, 36, 59). The proposed educational mapping enables learners to connect an observed disparity with possible mechanisms, health consequences and a justified response. It also provides educators with a basis for aligning activities and assessment.

**Table 3:** The five competency dimensions of the SBL-PHE Framework.

Dimension	Knowledge	Skills	Professional disposition
1. Recognize bias	Forms of selection, measurement, representation, label, proxy and implementation bias	Identify potential bias in evidence, datasets, cases, model documentation and outputs	Critical vigilance and willingness to question presumed neutrality
2. Explain bias	How bias arises across the sociotechnical AI lifecycle	Analyze technical, historical, institutional and professional mechanisms	Systems thinking and contextual interpretation
3. Evaluate bias	Effects on health equity, quality, resource allocation and group-specific errors	Assess fairness metrics, trade-offs, uncertainty and population-level consequences	Equity orientation and reasoned normative judgment
4. Address bias	Technical, organizational, participatory and governance responses	Develop context-sensitive criteria for mitigation, procurement, implementation, monitoring and escalation	Accountability and willingness to intervene or reject
5. Reflect on bias	Personal assumptions, epistemic limits, power relations and excluded knowledge	Examine one’s own judgment, role, trust and the limits of quantitative evidence	Critical humility and openness to contextual and experiential knowledge

Authors’ conceptual synthesis connecting lifecycle analysis, sociotechnical fairness, AI literacy and public health competencies (4, 14, 36, 59). The dimensions are interconnected and may be revisited throughout the case. The table specifies proposed educational objectives, not validated measures of competence.

Sociotechnical Bias Literacy provides the foundation for this reasoning. Sociotechnical Fluency describes a more advanced capacity to translate it into decisions across technical and professional settings. In the case below, recognizing that a model performs differently across groups is an expression of literacy. Coordinating an appraisal of the model with clinical expertise, service capacity and institutional responsibility illustrates the proposed fluency horizon. The distinction concerns the depth and integration of judgment within a professional role, rather than a separate sixth competency dimension or a requirement that every learner become a model developer.

### 4.2 Applying SBL-PHE to the cardiovascular teaching case

In the composite teaching case, a regional public health and healthcare network is considering a predictive AI system to inform invitations to preventive cardiovascular examinations. The system estimates future cardiovascular risk from routinely collected clinical and demographic data. Overall performance is reported as good, but subgroup information is limited. This is an illustrative design problem, not an evaluation of a commercial product. The proposed teaching materials combine published evidence with clearly labeled illustrative model documentation and scenarios.

The analysis begins with the knowledge underlying the data. Women have historically been underrepresented in cardiovascular trials (6). Reviews of acute coronary syndromes also show that chest pain is common in both women and men, while women more frequently present with symptoms such as nausea or vomiting, shortness of breath and pain between the shoulder blades (60, 61). Describing these presentations indiscriminately as atypical can obscure clinically relevant variation. This evidence motivates scrutiny of diagnostic expectations and documentation; the teaching system concerns preventive risk assessment, not triage of acute symptoms.

Learners examine whether the recorded data represent the population eligible for prevention and whether outcomes are observed consistently. In this scenario, delayed diagnosis or incomplete follow-up could affect whether cardiovascular disease enters the record. A utilization variable may reflect access to appointments, diagnostic testing or treatment as well as health need. Employment, unpaid care work and postcode may similarly capture social circumstances influencing both health and access to services. Such variables are neither inherently inappropriate nor automatically valid measures of disease risk. Their meaning depends on how they were recorded, the prediction target and the intended decision (3, 5).

An evidence and data audit brings these questions into a concrete task. Using a data dictionary and evidence extracts, learners compare the development and target populations, examine missingness, and distinguish recorded sex from information about gender-related circumstances. They assess which labels can support the intended prediction and identify gaps requiring further investigation. Their evidence-supported bias map links each concern to a possible mechanism and consequence, while distinguishing documented findings from hypotheses.

Numerical representation alone does not resolve the problem. In Li et al. (8), women comprised 64.5% of a cohort of 109,490 individuals, yet the evaluated cardiovascular prediction models showed disparities disadvantaging women. This finding supports examining feature distributions, outcome prevalence and model decisions alongside representation. It does not establish that any one of these factors caused the disparities in the teaching scenario. Mihan et al. (16) provide a broader discussion of bias in cardiovascular AI rather than an independent replication of that cohort study.

The next activity focuses on model development and validation. Learners review a Model Card describing intended use, predictors, outcomes and development and validation populations (47). A subgroup performance table allows comparison of sensitivity, specificity, positive predictive value, calibration and false-negative rates. Sample sizes and uncertainty accompany the comparisons so that an apparent difference is not automatically interpreted as a reliable disparity. The analytical memo explains which findings warrant further assessment and how they could affect invitations to preventive care.

SHAP or LIME outputs can complement this review by indicating which features contribute to model predictions (45, 46). For example, a strong contribution from healthcare utilization may prompt learners to ask whether the model is relying on patterns of service access rather than the intended disease risk. This is a hypothesis requiring further investigation. Feature attribution does not by itself establish that a feature is clinically invalid or that a model performs worse for women. Those questions require clinical appraisal, subgroup evaluation and, where appropriate, targeted testing for shortcut learning (27). Learners therefore use explanations to formulate testable concerns and interpret them alongside performance evidence.

The case then turns to the decision threshold and its consequences. Equal opportunity concerns comparable true-positive rates, demographic parity concerns comparable positive-decision rates, and predictive parity concerns comparable positive predictive values. These criteria answer different questions. With differing outcome prevalence, predictive parity generally conflicts with simultaneously equal false-positive and false-negative rates, subject to the mathematical assumptions and exceptional cases described by Chouldechova (44). Equal opportunity alone is a weaker requirement than equality of both error rates (43). Calibration across risk scores should also be distinguished from predictive parity at a selected threshold. Figure 3 summarizes the group fairness criteria considered in this discussion.

**Figure 3:**
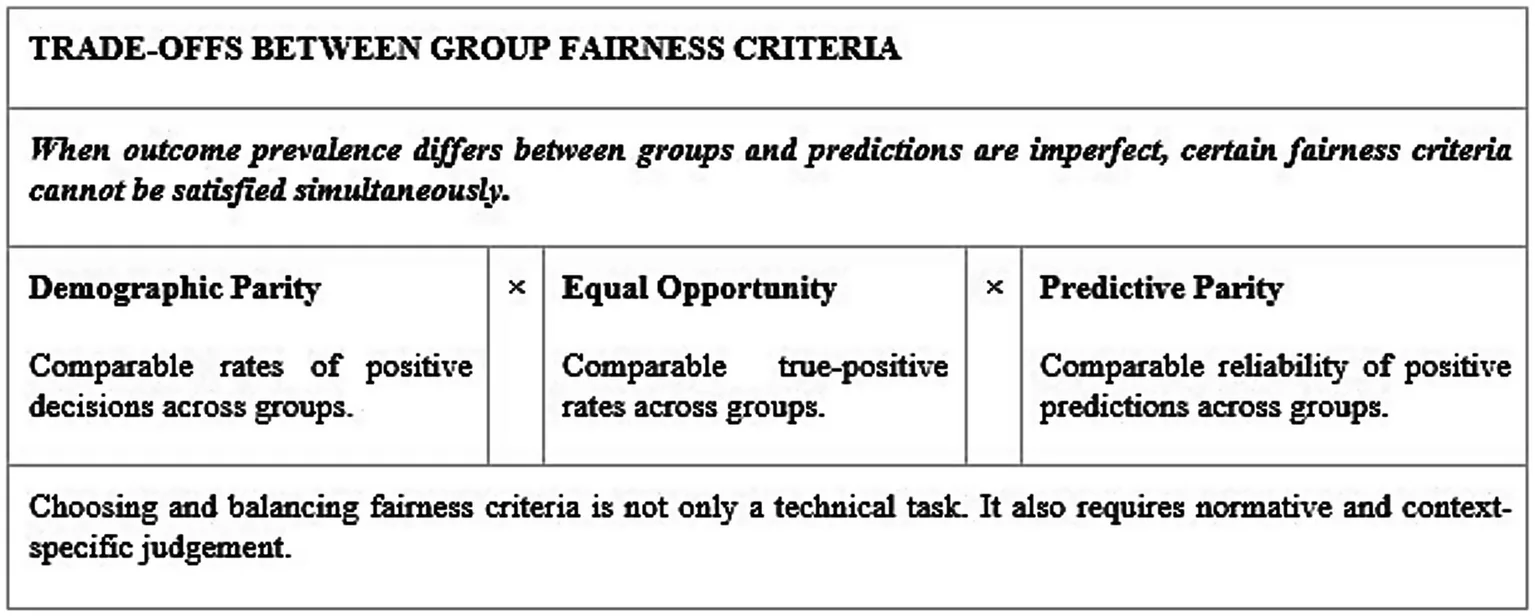
Trade-offs between group fairness criteria. Demographic parity, equal opportunity and predictive parity address different aspects of fairness. When outcome prevalence differs between groups, some fairness requirements cannot generally be satisfied simultaneously; the specific incompatibilities depend on the criteria and assumptions considered. Selecting and balancing criteria requires technical analysis and normative, context-specific judgment. Authors’ illustration based on Hardt et al. (43) and Chouldechova (44).

In an interprofessional case conference, learners consider a scenario in which a higher false-negative rate for women could lead to missed opportunities for prevention. They compare this with a lower threshold that would generate more invitations and potentially more unnecessary investigations. The decision brief specifies the public health objective, the fairness priority, likely benefits and burdens, uncertainty and implications for service capacity. The task requires a reasoned choice rather than the assumption that one fairness metric is appropriate in every setting (10).

Implementation extends the analysis from prediction to organizational action. A shared decision rule can repeatedly affect access to preventive services across sites and over time. Monitoring therefore needs to examine both model performance and the pathway from invitation to attendance, further assessment and care. The governance simulation brings these technical and organizational questions together. Learners decide whether introduction is justified, whether additional validation or modification is required, or whether use should be restricted or deferred. They specify responsibilities, monitoring intervals, escalation routes and conditions for suspending use (9, 13, 48).

The proposed monitoring approach extends beyond a binary comparison of women and men. It considers how sex- and gender-related circumstances interact with age, socioeconomic position, disability and barriers associated with racialization or migration (34, 35). Small subgroups may produce imprecise estimates, and relevant information may be unavailable. These limitations should remain visible in the assessment. Stakeholder accounts and incident reports can complement quantitative analysis when performance estimates alone are insufficient. Human oversight is thus treated as an institutionally supported capacity to act. Table 4 summarizes the integrated sequence of technical scrutiny, learning activities, proposed materials and assessment.

**Table 4:** Integrated analytical and learning sequence for the cardiovascular teaching case.

Phase	Technical object of scrutiny	Learning activity	Proposed materials	Assessment
Knowledge and data	Representation, measurement, missingness and labels	Evidence and data audit	Case description, evidence extracts and data dictionary	Evidence-supported bias map
Model	Objective function, thresholds, validation and subgroup performance	Model Card review and metric comparison	Illustrative Model Card, subgroup performance table and optional feature-attribution output	Brief analytical memo
Output and decision	Fairness criteria, error distributions and distributional consequences	Interprofessional case conference	Threshold scenario and stakeholder positions	Justified decision brief
Implementation and monitoring	Local validation, drift, auditing and human oversight	Governance and monitoring simulation	Monitoring template and role descriptions	Implementation and escalation plan

Authors’ proposed teaching design informed by the cardiovascular, fairness and auditing literature cited in section 4.2. Materials and assessments illustrate how the framework could be implemented. Illustrative model information must be distinguished from findings reported in the source studies. Assessments provide opportunities to demonstrate the five SBL-PHE competency dimensions within the case.

### 4.3 Curricular integration and prospective evaluation

An initial allocation of six to eight contact hours is proposed for the case sequence, with its feasibility to be examined during piloting. The sequence can be embedded in existing courses on research methods, epidemiology, health promotion, health economics, ethics or health policy. Shared competency objectives connect activities that might otherwise remain separated across disciplines. Technical programs may extend the case through data analysis, while other programs can work with prepared documentation and performance summaries.

Constructive alignment connects the intended competencies with activities that elicit them and assessment tasks that make the reasoning observable (56). Krathwohl’s (57) distinction between types of knowledge supports attention to conceptual, procedural and metacognitive learning. Miller’s (58) assessment framework distinguishes knowledge and demonstrated performance from action in professional practice. An evidence audit or decision brief therefore provides evidence about learning in the educational setting, while professional transfer requires additional investigation. Learners practice requesting relevant technical assessments, interpreting findings and identifying when specialist expertise is needed (9, 50).

The framework and its educational implementation have not yet been empirically evaluated. A prospective mixed-methods pilot could examine feasibility, acceptability and learning through scenario-based assessments, observations and interviews with learners and educators. Feasibility measures would include time requirements, task completion and the usability of materials. Interviews would examine whether the activities are understandable, relevant and appropriate to learners’ prior knowledge and roles.

A pre–post assessment could use parallel cases of comparable difficulty and a rubric aligned with the five competency dimensions. Assessment would examine whether learners identify plausible bias sources, explain mechanisms, interpret subgroup metrics, justify a fairness priority and propose appropriate mitigation, monitoring or escalation. Reflection would be assessed through the explicit consideration of uncertainty, assumptions and professional responsibility. Self-reported confidence could supplement these tasks but would not serve as the sole evidence of learning. Independent scoring of a subset could examine inter-rater agreement. Rubric validity, sensitivity to change and transfer to a later unfamiliar or practice-based task would require empirical evaluation. A pilot without a comparison group could inform refinement but would not establish that the framework caused any observed improvement.

## 5 Discussion

SBL-PHE connects sociotechnical analysis of the AI lifecycle with five competency dimensions, a continuous teaching case and proposed curricular activities and assessment. Its contribution lies in specifying how public health learners can connect technical evidence about algorithmic bias with health equity, professional judgment and institutional responsibility. The framework offers a basis for educational design; whether this design improves learning or practice remains an empirical question.

This contribution requires conceptual clarity because bias denotes different phenomena in machine learning, statistics, clinical judgment and research on justice. Inductive bias comprises the assumptions and preferences that enable a learning algorithm to generalize beyond observed data. Finite observations alone do not uniquely determine predictions for unseen cases. Assumptions about the hypothesis space or preferences between possible solutions therefore shape learning, but their presence does not in itself establish discrimination (19).

Inductive bias must also be distinguished from bias in the classical bias–variance decomposition of squared prediction error. Here, bias concerns the systematic difference between the expected prediction across training samples and the target function. Greater model flexibility can reduce this component while increasing variance and susceptibility to overfitting. This trade-off concerns predictive behavior, not whether a system distributes benefits and harms fairly (62).

Clinical heuristics similarly require a differentiated assessment. Simple decision strategies can support efficient judgment under uncertainty, although their usefulness depends on the task and environment. Heuristics are not synonymous with cognitive errors, but they can lead to systematic errors under particular conditions (63, 64). Human oversight must therefore examine the conditions supporting professional judgment rather than assuming that the presence of a person automatically corrects algorithmic limitations.

Responsible AI consequently requires a more precise objective than undifferentiated freedom from bias. Model assumptions and distinctions between groups need to be assessed against their intended purpose, available evidence and consequences. The concern is with harmful mechanisms such as inadequately justified proxies, distorted labels and unequal care conditions that become embedded in apparently neutral outputs (5, 10, 65). These mechanisms must be established empirically rather than inferred from a disparity alone. Obermeyer et al. (66), for example, identified healthcare expenditure as a problematic proxy for health needs in an algorithm disadvantaging Black patients. Li et al. (8) reported disadvantages affecting women despite their numerical majority in the cardiovascular cohort. The latter finding challenges an exclusively numerical account of representation; it does not establish that the cost-proxy mechanism identified by Obermeyer et al. explains the gender disparities.

The public health perspective extends this analysis from individual predictions to repeated decisions across populations. Standardized rules can produce cumulative differences in prevention, care and resource allocation. Responsibility therefore encompasses the organizations that procure, validate, implement and monitor systems, alongside those who develop or use them (5, 9). Educational competence alone cannot compensate for an absence of time, authority or procedures for challenging an output. Effective oversight requires institutional arrangements through which concerns can lead to investigation, modification or suspension of use (13).

The cardiovascular teaching case brings these relationships into a common decision setting. It connects evidence gaps and documentation practices with model performance, fairness criteria and access to prevention. Subgroup metrics can reveal disparities, while feature-attribution methods and audits can support further investigation. None of these tools can determine which errors are acceptable or how benefits and burdens should be distributed. The incompatibility of some fairness criteria under specified conditions makes explicit justification particularly important (44). Within SBL-PHE, learners are therefore asked to explain a fairness priority in relation to public health objectives, affected populations and service conditions. Participation by affected groups, documented responsibilities and criteria for escalation are integral to the proposed decision process.

Monitoring after implementation extends this responsibility over time. Changes in population composition, documentation and care pathways can alter model performance and the consequences of its use (9, 48). Sun et al. (67) evaluated FairICP using medical imaging datasets, applying subpopulation-specific conformal calibration to identify uncertain predictions and support abstention. This illustrates a potential technical contribution to monitoring, with results dependent on calibration data and the balance between retained predictions and abstention. It does not establish long-term clinical benefit or replace local assessment. For teaching, it also raises a practical question about what happens to people whose predictions are withheld and whether the subsequent assessment pathway introduces further inequalities.

Distinguishing Sociotechnical Bias Literacy from Sociotechnical Fluency helps align these demands with professional roles. Literacy provides the foundation for recognizing limitations, asking informed questions and interpreting evidence. Public health professionals need not conduct every technical audit themselves, but they should understand its purpose, findings and limits sufficiently to judge when specialist support is needed. Fluency extends this foundation through the ability to connect technical, clinical and institutional perspectives in implementation, evaluation and governance. It is an advanced, role-dependent educational horizon rather than an identical requirement for all graduates. This distinction supports integration into existing teaching in epidemiology, research methods, health promotion, health economics and health policy. Universities and public health organizations should also apply the standards they teach to their own procurement and use of AI.

Several limitations qualify these contributions. The framework has not been empirically validated, and the proposed competency dimensions, assessment rubric and curricular sequence require operationalization and testing. The focused supplementary search supported conceptual refinement rather than exhaustive retrieval or effect estimation. Targeted extraction from 26 core sources does not constitute completed screening of the larger consolidated search corpus. The source selection and conceptual mapping involve interpretive judgments, and AI-assisted processing does not provide independent duplicate screening or extraction. Neither the size of the retrieved corpus nor the availability of an evidence matrix establishes comprehensive coverage or conceptual saturation.

The cardiovascular case is evidence-informed but composite and does not evaluate a single deployed system. Its emphasis on sex and gender is intended to make mechanisms concrete, but findings from particular populations cannot be assumed to apply uniformly across settings. Analyses must also consider variation within groups and the interaction of gender with socioeconomic position, age, racialization and other conditions shaping health and access to care (3, 35). The feasibility of such assessment depends partly on the availability and quality of relevant data.

Future research should examine the framework across degree programs, qualification levels and professional roles. Initial pilots can assess feasibility, acceptability and the clarity of assessment criteria. Subsequent evaluation should examine whether learners can transfer their reasoning to unfamiliar cases, manage uncertainty and challenge inadequately justified outputs. Longer-term work should consider professional judgment and institutional action alongside knowledge gains, including the organizational conditions that enable learning to influence practice.

## 6 Conclusion

Algorithmic bias is a public health challenge shaped by the interaction of historical evidence, data practices, model development, institutional routines and societal power relations. Addressing harmful bias requires technical scrutiny alongside professional judgment and institutional accountability. Neither technical optimization nor the formal presence of a human in the decision loop is sufficient.

The SBL-PHE Framework translates this sociotechnical perspective into five interconnected competency dimensions concerned with recognizing, explaining, evaluating, addressing and reflecting on algorithmic bias. Its proposed educational application connects learning objectives, activities and assessment through an evidence-informed composite cardiovascular case. The case illustrates how sex and gender inequalities can be examined across the AI lifecycle, including their interaction with other conditions shaping health and access to care.

Sociotechnical Bias Literacy provides the proposed foundation for professionals who use or interpret AI-supported knowledge. Sociotechnical Fluency extends this foundation toward an advanced, role-dependent capacity to connect technical evidence with professional, institutional and societal considerations. SBL-PHE offers educators a conceptual basis for integrating these competencies into existing public health curricula. Empirical evaluation is needed to establish its educational value and the conditions under which learning translates into responsible practice.

## Data Availability

The original contributions presented in this study are included in the article. Further inquiries can be directed to the corresponding author.
